# Effects of exercise on secretion transport, inflammation, and quality of life in patients with noncystic fibrosis bronchiectasis: Protocol for a randomized controlled trial

**DOI:** 10.1097/MD.0000000000009768

**Published:** 2018-02-16

**Authors:** Daniele Oliveira dos Santos, Hugo Celso Dutra de Souza, José Antônio Baddini-Martinez, Ercy Mara Cipulo Ramos, Ada Clarice Gastaldi

**Affiliations:** a Department of Physiotherapy; b Internal Medicine Department, Ribeirão Preto Medical School, University of São Paulo; c Department of Physiotherapy, São Paulo State University, Presidente Prudente, São Paulo, Brazil.

**Keywords:** bronchiectasis, clinical trial, exercises, inflammation, mucociliary clearance, rehabilitation

## Abstract

**Background:**

Bronchiectasis is characterized by pathological and irreversible bronchial dilatation caused by the inefficient mucus and microorganism clearance and progression of inflammatory processes. The most frequent characteristic is the increase in bronchial mucus production resulting in slower transport and damage to the mucociliary transport.

**Aims:**

To evaluate the effects of exercise on mucus transport, inflammation, and resistance of the respiratory and autonomic nervous systems and subsequent effects on quality of life in patients with bronchiectasis who are enrolled in a pulmonary rehabilitation program.

**Methods:**

Sixty subjects of both sexes between 18 and 60 years (30 volunteers with clinically stable bronchiectasis and 30 healthy volunteers) will be included. Participants with chronic obstructive pulmonary disease, decompensated cardiovascular or metabolic diseases, neuromuscular and musculoskeletal diseases, and active smokers will be excluded. Volunteers will be randomly allocated to the pulmonary rehabilitation or control groups. The primary outcomes will be nasal transport time as evaluated by nasal saccharin transport time, analysis of nasal lavage, enzyme immunoassay of exhaled expiration, and analysis of the mucus properties. The secondary outcomes will include pulmonary function tests, impulse oscillometry, heart rate variability analysis, and quality of life questionnaires.

**Discussion:**

In addition to the benefits for patients already described in the literature, the additional benefit of mucus removal may contribute to optimizing treatments and better control of the disease.

**Conclusion:**

This protocol could provide new information about the unclear mechanisms regarding exercise to aid in the removal of secretions.

## 1 Introduction

Bronchiectasis is characterized by pathological and irreversible bronchial dilatation.^[1,2]^ It is mainly caused by inefficient removal of secretions and microorganisms, as well as the perpetuation of inflammatory processes induced by chronic or recurrent infections. The diagnosis is traditionally performed using high-resolution computed tomography (HRCT) in association with spirometry.^[3,4]^

Spirometry requires great cooperation from subjects. Alternative tests such as impulse oscillometry system (IOS) are suggested for patients who have difficulties in performing the forced manoeuvres.^[5]^ IOS evaluates the impedance of the respiratory system by applying oscillatory pressure waves at multiple frequencies. This technique has been shown to be more sensitive in the identification of airway alterations than spirometry.^[6,7]^ Clinically, the most frequent characteristic is an increase in the production of bronchial secretions. The mucus produced is thicker and results in slower transport, causing damage to the mucociliary transport.^[8]^ Patients may undergo exacerbation periods and acute deterioration of respiratory symptoms related to decreased quality of life, lung function, and increased mortality.^[4]^

The immune response in bronchiectasis is primarily mediated by neutrophils, and increased levels of chemokines and proinflammatory cytokines are found in the airways of affected individuals.^[8]^ High levels of proteases are present at inflammation sites, resulting in the release of proinflammatory cytokines. This causes even more damage to the cells of airway structures.^[9]^ In approximately 50% of cases, there is a diagnosis of a primary disease causing bronchiectasis.^[10]^ Thus, a single clinical or functional parameter is insufficient to assess the severity of the disease. In an attempt to elucidate this problem, Chalmers et al^[11]^ developed the Bronchiectasis Severity Index and concluded that it more accurately stratifies mortality risk, hospital admissions, and future risk of exacerbations. Patients may benefit from specific physiotherapy techniques for the removal of secretions from the respiratory system, as well as overall exercises within pulmonary rehabilitation programs.^[12]^ However, there is little information on the effects of physical training in patients with bronchiectasis.

Several factors induce dysautonomia in patients with bronchiectasis, with probable sympathetic hyperactivation representing an important cardiovascular risk.^[13]^ Impaired gas exchange has been associated with increased sympathetic activation.^[14]^ The autonomic nervous system acts on the mucociliary clearance system, but autonomic nervous system dysfunction in bronchiectasis has been poorly studied. Analyzing heart rate variability may be an excellent measure of autonomic regulation.^[15]^

This study evaluates the effects of exercise on patients with bronchiectasis in a pulmonary rehabilitation program. The considerations include the transport of secretions, inflammation and resistance of the respiratory system, nervous system autonomy, and the repercussions on the quality of life.

## 2 Methods

### 2.1 Ethical approval

Ethical approval was obtained on March 16, 2016, by the Research Ethics Committee of the Clinical Hospital of the Ribeirão Preto Medical School at the University of São Paulo (2053/2016). The trial has been prospectively registered at ClinicalTrials.gov (NCT02823587). Once patients have accepted an invitation to participate in the trial, their written consent will be obtained before assessing them for eligibility by D.O.S.

### 2.2 Study design

The trial is prospective, single-center, parallel, randomized, assessor-blinded trial. This trial will follow the recommendations of the Consolidated Standards of Reporting Trials statement^[16]^ and the Standard Protocol Items for Randomized Trials.^[17]^

### 2.3 Recruitment organization and randomization

**Figure 1:**
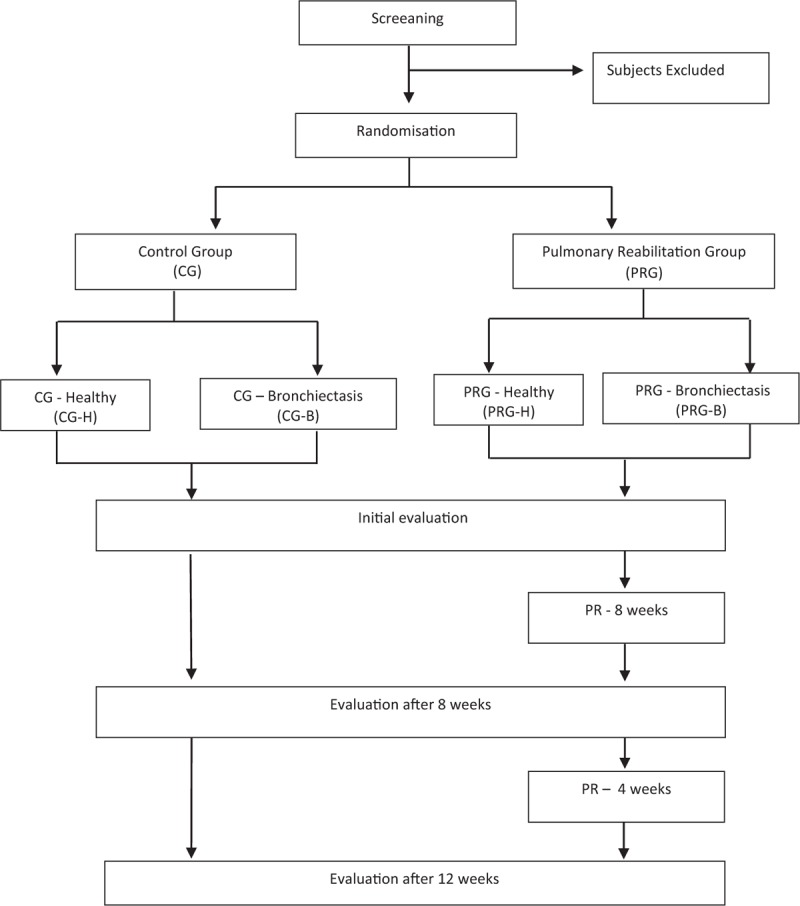
Study design. Flow of participants according to the Consolidated Standards of Reporting Trials (CONSORT).

The volunteers will be recruited in the pulmonology outpatient of Clinical Hospital of the Ribeirão Preto Medical School. A pulmonologist will evaluate HRCT results to determine eligibility. Only volunteers who satisfy all the criteria inclusion will be randomized using simple computerized randomization procedures. After, it will be divided into a pulmonary rehabilitation group and a control group (CG). These groups will be subdivided into a group that will be instructed to follow an exercise protocol and one that will not (Fig. 1). The allocation sequence will be generated by a researcher who is not involved in the assessment and interventions. After allocation to one of the groups, a blind examiner will conduct clinical evaluations and collect data to analyze the primary and secondary variables. Another evaluator will follow the exercise plan. All participants will be assessed at the start of the study after the 8th and 12th weeks. Volunteers may discontinue participation at any time if they wish. However, all efforts will be made to avoid missing data, mainly for the main target criteria, case a patient discontinues the trial, a final visit will be performed, and the data of this visit will be used for the calculation of the main criteria, the primary outcomes. The specific dealing of missing data will be determined in a data review meeting before starting statistical analyses.

### 2.4 Study participants and eligibility criteria

A total of 60 eligible participants with age between 18 and 60 years will be recruited. Of these participants, 30 will have been diagnosed with bronchiectasis that is not attributable to cystic fibrosis and confirmed by HRCT. These subjects will be clinically stable without any evidence of exacerbated bronchiectasis or changes in medical therapy in the previous 4 weeks.^[18]^ Participants will be excluded if they have a smoking history ≥10 pack years, diagnosis of chronic obstructive pulmonary disease,^[19]^ asthma,^[20]^ interstitial lung disease (clinical/radiological diagnosis), and medical conditions that can put the subjects at risk during the exercise protocol (eg, unstable cardiovascular disease) or conditions that may restrict the participant's ability to exercise (eg, severe orthopedic or neurologic impairments). The remaining participants will be 30 nonsmoker volunteers without lung disease and recruited from the community.

### 2.5 Intervention

#### 2.5.1 Intervention group (pulmonary rehabilitation group)

The intervention group will participate in a supervised exercise program twice a week for 12 weeks. This program will consist of an individual prescription of exercise on a treadmill or bicycle at an initial intensity of 80% of the maximal oxygen consumption (VO_2_ max)^[21]^ obtained from a cardiopulmonary test, as well as active or active-resistance exercises for the upper and lower limbs. The exercises will be performed according to the perception of effort according to the modified Borg scale.^[22]^ Adverse event will be defined as any change in mean arterial blood pressure higher then >140 or less then <65 mm Hg, heart rate <50 or >140 bpm, arrhythmias with hemodynamic consequences, myocardial ischemia, and SpO_2_ <88%. If any adverse event happen, the session will be interrupted the event will be related. A home exercise program will also be prescribed with the goal of achieving 3 to 5 unsupervised sessions per week with the aim of incorporating a regular routine of moderate-intensity physical exercise into the daily lives of the volunteers. The program will consist of 45 minutes of walking (using the Borg scale to reach the desired intensity). This activity will be recorded by a researcher in an individual diary for each volunteer. Participants will be instructed to maintain the exercise routine throughout the study period and will be reminded weekly through telephone calls.

#### 2.5.2 Control group (CG)

Participants in the CG will not participate in supervised exercise sessions. They will only be informed at the beginning of the study that performing 30 minutes of moderate-intensity physical activity several days a week is associated with health benefits. During the study, participants in this group will be contacted monthly by phone to provide general support and counseling without discussing exercise or physical activity.

### 2.6 Outcomes

#### 2.6.1 Primary outcomes

##### 2.6.1.1 Saccharin transport time

It is a nasal technique for to measure mucociliary clearance, widely reported as a simple, non-invasive, valid and reproducible method.^[23]^ The nasal cavity has a respiratory epithelium similar to the rest of the respiratory tract, and nasal mucociliary transport has a good correlation with tracheobronchial transport.^[24]^ Subjects will be seated and positioned with 10 degrees of neck extension. Granulated sodium saccharin (250 μg) will be placed 2 cm inside the right nostril under visual control. The time from particle placement until the first perception of a sweet taste in the mouth will be recorded in minutes by a digital chronometer.

##### 2.6.1.2 Secretion analysis

Mucus samples will be collected by voluntary coughing and expectoration into a glass. The mucus will be separated from the saliva and then evaluated in terms of its adhesiveness and purulence. After this initial evaluation, the respiratory secretions will be placed in plastic tubes and covered with mineral oil to prevent dehydration, followed by freezing for subsequent analysis.^[25]^ The evaluated parameters will be the surface properties, appearance, relative transport velocity on a frog palate, transport measured in a simulated cough machine, and the contact angle.^[26]^

##### 2.6.1.3 Nasal lavage fluid

In a sitting position, participants will be instructed to tilt the head back at a 45° angle and close the nasopharynx with the soft palate. Five milliliters of 0.9% saline solution at room temperature will be instilled into each nostril, and subjects will be instructed to hold their breath without swallowing for 10 seconds. After 10 seconds, the individuals will blow their nose hard into a sterile plastic tube. The samples will be centrifuged (10 minutes, 5 °C), and the supernatant will be separated from the pellet and immediately transferred to sterile polypropylene tubes. The tubes will be coded and stored at −80 °C for determination of the cytokine levels.^[27]^

##### 2.6.1.4 Exhaled breath condensate collection and pH analysis

It is difficult to obtain specimens noninvasively from the lower airways to assess inflammation. However, the exhaled breath condensate provides information about the lower to upper airways and can be performed repeatedly. The collection will be performed as described previously.^[28]^ The samples (2.0–2.5 mL) will immediately be divided into 500-μL aliquots and transferred to sterile polypropylene tubes, which will be encoded and stored for a maximum of four weeks at −80 °C for the determination of the cytokine levels. The pH of the samples will be measured immediately, and then the measured samples will be discarded.

##### 2.6.1.5 Measurements of the nasal fractional exhaled nitric oxide

The nasal fractional exhaled nitric oxide level will be measure using a hand-held chemiluminescence analyzer (NIOX MINO Airway Inflammation Monitor, Aerocrine AB, Solna, Sweden). Three acceptable measures and mean values will be included in the analysis. The measurement range will be 5 to 400 ppb. The measurements will be performed according to a protocol defined by the American Thoracic Society.^[29]^

##### 2.6.1.6 Cytokine analysis

The concentrations of tumor necrosis factor-alpha, interleukins 6 and 10 in nasal lavage fluid samples will be determined using high-sensitivity immunoassay (ELISA) performed according to the instructions of the supplier of the kit.

#### 2.6.2 Secondary outcomes

##### 2.6.2.1 Pulmonary function

Pulmonary function will be evaluated by Koko PFT System Spirometer (version 4.11, 2007 nSpire Health, Inc., Pulmonary Data Services). The forced expiratory volume in the first 2nd and forced vital capacity will be determined according to the guidelines from the American Thoracic Society/European Respiratory Society Task Force.^[30]^ The percentages of predicted spirometry values will be calculated from published Brazilian population data.^[31]^

##### 2.6.2.2 Impulse oscillometry

Impulse oscillometry will be performed using a Jaeger IOS (Jaeger, Wurzburg, Germany).^[32,33]^ IOS parameters will be recorded, including respiratory impedance (Z), airway resistance at 5 Hz (R5), airway resistance at 20 Hz (R20), lung resistance at 5 Hz (X5), resonance frequency (Fres), and reactance area. It will enable analysis of the central and peripheral resistance of the respiratory system.

##### 2.6.2.3 Respiratory muscle strength test

The respiratory muscle strength will be assessed using a previously calibrated digital manovacuometer (MVD 300, Globalmed, Porto Alegre, Brazil) with a variation of ±300 cm H_2_O. First, maximal inspiratory pressure will be measured from the residual volume, followed by maximal expiratory pressure from the total lung capacity. The test will be performed according to the Guidelines for Pulmonary Function Testing.^[31,34]^

##### 2.6.2.4 Quality of life evaluation scale

The general health status of the patients will be assessed through the Medical Outcomes Study 36-Item Short-Form Health Survey.^[35]^ The quality of life in this group of patients will be evaluated using the Quality of Life in Bronchiectasis Questionnaire^[11]^ and the Leicester Cough Questionnaire to assess the physical, psychological, and social impacts of chronic cough.^[36]^

##### 2.6.2.5 Analysis of heart rate variability

Heart rate variability will be assessed by spectral analysis using echocardiogram records.^[15]^ Participants will be placed in the supine position for 20 minutes on an electric orthostatic table. They will then be passively placed in a tilted position at 75° for another 20 minutes (tilt test). Electrocardiogram measurements (ML866 PowerLab, ADInstruments, Bela Vista, NSW, Australia) will be obtained in the lying and supine positions and during the tilt test. The respiratory rate will be set at 15 cycles per minute with the aid of a metronome.

#### 2.6.3 Psychosocial measures

##### 2.6.3.1 Minimental state examination

The minimental state examination is commonly used as a screening test for dementia.^[37]^ All volunteers will undergo this assessment to screen for cognitive losses that may compromise the answers to the quality-of-life questionnaires. The maximum score is 30. Scores less than 24 are used by some researchers and clinicians to indicate possible dementia.

##### 2.6.3.2 Hospital anxiety and depression scale

The Hospital Anxiety and Depression Scale (HADS)^[38]^ is used to identify anxiety disorders and depression in physically ill patients. The HADS is divided into an anxiety subscale (Hospital Anxiety and Depression Scale-Anxiety Subscale) and a depression subscale (Hospital Anxiety and Depression Scale-Depression Subscale), both of which contain 7 interspersed items. The cut-off scores are 8 points for anxiety and 9 for depression.

### 2.7 Sample size calculation

The sample calculation was performed based on the mucociliary clearance evaluated by the saccharine transport time.^[39]^ The detected effect should have an average of 4 minutes with a standard deviation of 3 minutes, alpha of 5%, and power of 95%. The results indicate that 60 volunteers would be needed with 15 in each subgroup.

### 2.8 Statistical analysis

A biostatistician will analyze the data in a blinded manner. The normality of the data will be verified by the Shapiro–Wilk test. For comparing the values between 2 groups, the Student *t* test will be used for parametric variables and the Wilcoxon test will be used for nonparametric variables. Analysis of variance will be used for comparison between 3 or more groups, followed by a multiple comparisons test when indicated for parametric data or the Kruskal–Wallis test for nonparametric data. Data correlation will be assessed using the Pearson or Spearman correlation test for normal and nonnormal distributions, respectively. The level of significance will be set at 5%. Statistical analysis will be performed through the R version 3 (2013-05-16) (The R Foundation for Statistical Computing, Vienna, Austria) and SAS version 9.2 (Cary, NC).

### 2.9 Data management

All data will be entered electronically. There will be a subject identification code list and each participant will receive a participant study identification number. Confidential documents will be retained in the institution's computer in the laboratory. A password system will be utilized to control access. Back up of data will be kept in locked cabinets. Any modifications to the protocol and administrative changes of the protocol will be communicated to ClinicalTrials.gov and Research Ethics Committee and clinical research unit of the Hospital das Clínicas da Faculdade de Medicina de Ribeirão Preto. All principal investigators will have access to the final trial dataset.

## 3 Discussion

The main problem for bronchiectasis patients is the retention of secretions^[1]^ associated with bacterial colonization and inflammation, with frequent exacerbations by infection. These cycles can contribute to the progression of the disease. Patients with bronchiectasis present reduced exercise capacity associated with structural changes in lung tissue and progressive airflow obstruction. In the more advanced phases of the disease, patients can present static and dynamic hyperinflation, leading to a decrease in tidal volume along with increases in dead space ventilation and effort-related dyspnea.^[40–44]^ These factors lead to a sedentary lifestyle, which affects daily life activities and worsens quality of life.

International guidelines recommend pulmonary rehabilitation programs for these individuals to improve exercise capacity through the effects on aerobic capacity and peripheral musculature, as well as improving disease control and quality of life.^[45]^ There is little information about the effects of physical training on patients with bronchiectasis. However, it is likely that the benefits of physical training in these patients are at least comparable to those demonstrated in other clinical conditions with respiratory dysfunction.^[21,46,47]^ Prospective studies about the effects of pulmonary rehabilitation on bronchiectasis patients have shown improvements in exercise tolerance and quality of life in the short term.^[21,36,48]^ Improvements were observed in the exacerbation frequency and time of the first exacerbation in the long term.^[36]^ Retrospective studies have demonstrated positive effects on exercise capacity, quality of life, and pulmonary function.^[46,49]^

The benefits of exercise have been demonstrated for secretions clearance in other respiratory diseases,^[50]^ as well as for reductions in mucus viscosity and elasticity.^[51–54]^ In subjects with cystic fibrosis, greater mucus expectoration after exercise has been reported. This effect may be due to increased ventilation and respiratory flow. The same study also observed that exercise on a treadmill reduced the mechanical impedance of the mucus, probably due to trunk oscillations associated with walking.^[55]^

Regular physical training can modify the autonomic balance and may accelerate the physiological recovery of the vagal sympathetic interaction.^[56]^ The autonomic nervous system acts on mucociliary clearance. Patients subjected to lung transplantation present a reduction in mucociliary clearance due to denervation since the vagus nerve mediates the main components of the mucociliary clearance system, the frequency of ciliary beats, and mucus secretion.^[57,58]^

The present study could provide new information about the unclear mechanisms regarding exercise to aid in the removal of secretions. We expect to identify whether mucociliary transport dysfunctions occur through only the oscillations promoted by increased airflow and body movement during exercise, or whether there are other mechanisms related to pulmonary mechanics, inflammation, or the autonomic nervous system.

## Acknowledgments

The authors thank the São Paulo Research Foundation for their financial support and Laboratory of Assessment Respiratory for equipment available for this study.
